# Pancreatic cancer: Emerging field of regulatory B-cell-targeted immunotherapies

**DOI:** 10.3389/fimmu.2023.1152551

**Published:** 2023-03-23

**Authors:** Zeynep Nur Senturk, Isilay Akdag, Bahar Deniz, Ayca Sayi-Yazgan

**Affiliations:** Department of Molecular Biology and Genetics, Istanbul Technical University, Istanbul, Türkiye

**Keywords:** pancreatic ductal adenocarcinoma (PDAC), tumor microenvironment (TME), regulatory B (Breg) cells, immunotherapy, interleukin 35 (IL-35)

## Abstract

Pancreatic ductal adenocarcinoma (PDAC), the most common type of pancreatic cancer, is characterized by a high mortality rate and poor prognosis. Current treatments for PDAC, are ineffective due to a prominent immunosuppressive PDAC tumor microenvironment (TME). Although B lymphocytes are highly infiltrated into PDAC, the importance of B lymphocytes in tumorigenesis is largely neglected. B cells play a dual role in the PDAC tumor microenvironment, acting as either anti-tumorigenic or pro-tumorigenic depending on where they are localized. Tumor-infiltrating B cells, which reside in ectopic lymph nodes, namely tertiary lymphoid structures (TLS), produce anti-tumor antibodies and present tumor antigens to T cells to contribute to cancer immunosurveillance. Alternatively, regulatory B cells (Bregs), dispersed inside the TME, contribute to the dampening of anti-tumor immune responses by secreting anti-inflammatory cytokines (IL-10 and IL-35), which promote tumor growth and metastasis. Determining the role of Bregs in the PDAC microenvironment is thus becoming increasingly attractive for developing novel immunotherapeutic approaches. In this minireview, we shed light on the emerging role of B cells in PDAC development and progression, with an emphasis on regulatory B cells (Bregs). Furthermore, we discussed the potential link of Bregs to immunotherapies in PDAC. These current findings will help us in understanding the full potential of B cells in immunotherapy.

## 1 Introduction

Pancreatic cancer is a highly aggressive, lethal malignancy, with a 5-year overall survival rate of 6-8% (1, 2). Most research on pancreatic cancer concentrates on pancreatic ductal adenocarcinoma (PDAC), which makes up roughly 90% of pancreatic cancers. PDAC is an exocrine tumor that occurs in the pancreatic ducts. The well-known risk factors for PDAC development include alcohol and tobacco use, ethnicity, type 2 diabetes, obesity, blood groups, microbiome composition, infections, and inherited germline mutations (3, 4).

PDAC is currently treated with resection surgery, chemotherapy, radiation, and immunotherapy. Overall, using the current treatment strategies has multi-faced limitations. Most patients who undergo surgical resection later relapse. PDAC is associated with stroma-rich desmoplastic and hypoxic environments that limit the efficacy of chemotherapy and radiotherapy (5, 6). Particular microbial species reside in PDAC tissues, which attenuate PDAC response to chemotherapeutic drugs (7–10). However, gut microbiome-induced high tumor microbial diversity promotes the long-term survival of patients with PDAC (11). As a result, there is still no agreement on whether the microbiome is tumorigenic or not.

The tumor microenvironment (TME) of PDAC lacks immunogenicity with inadequate immune activation and excessive immune suppression (10). While, CD8^+^ T cells, Th1- type CD4^+^ T cells, and natural killer (NK) cells exhibit anti-tumor activity in PDAC- TME, a strong immunosuppressive niche is established by regulatory T (Treg) cells, tumor-associated macrophages (TAMs), myeloid-derived suppressor cells (MDSCs), and their associated molecules. The ineffectiveness of immunotherapeutic approaches for treating PDAC is a result of this highly immunosuppressive environment (12).

Recently, PDAC immunotherapy approaches have focused on CD40 stimulation, targeting extracellular matrix components (ECM), CAR-T cell therapy, vaccination, and monoclonal antibodies (mAbs) against immune checkpoint receptors (13–18). Among these treatments, immune checkpoint blockade by immune checkpoint inhibitors and T-cell immunotherapies has shown effectiveness in fewer than 2% of PDAC patients (1, 19). The current immunotherapies for PDAC focus on decreasing immunotherapy resistance and boosting anti-tumor T-cell responses in TME, but they ignore the influence of B cells that are highly infiltrated into PDAC tissues. The PDAC-TME is inhabited by B-cell subsets with either pro-tumorigenic or anti-tumorigenic properties. Anti-tumorigenic B cells have a strong tumor suppressive activity by presenting tumor antigens to T cells and producing anti-tumor antibodies (IgG1), but specific pro-tumorigenic B lymphocyte subsets, namely, regulatory B (Breg) cells secrete immunosuppressive cytokines such as IL-10 and IL-35 thereby promoting cancer progression and evading immunosurveillance in PDAC (20).

In this review, we highlighted the emerging role of B cells, including Bregs, in the development of PDAC. Furthermore, we discuss the potential B cell-targeted immunotherapies for treating PDAC.

## 2 Roles of B cells in PDAC

**Figure 1:**
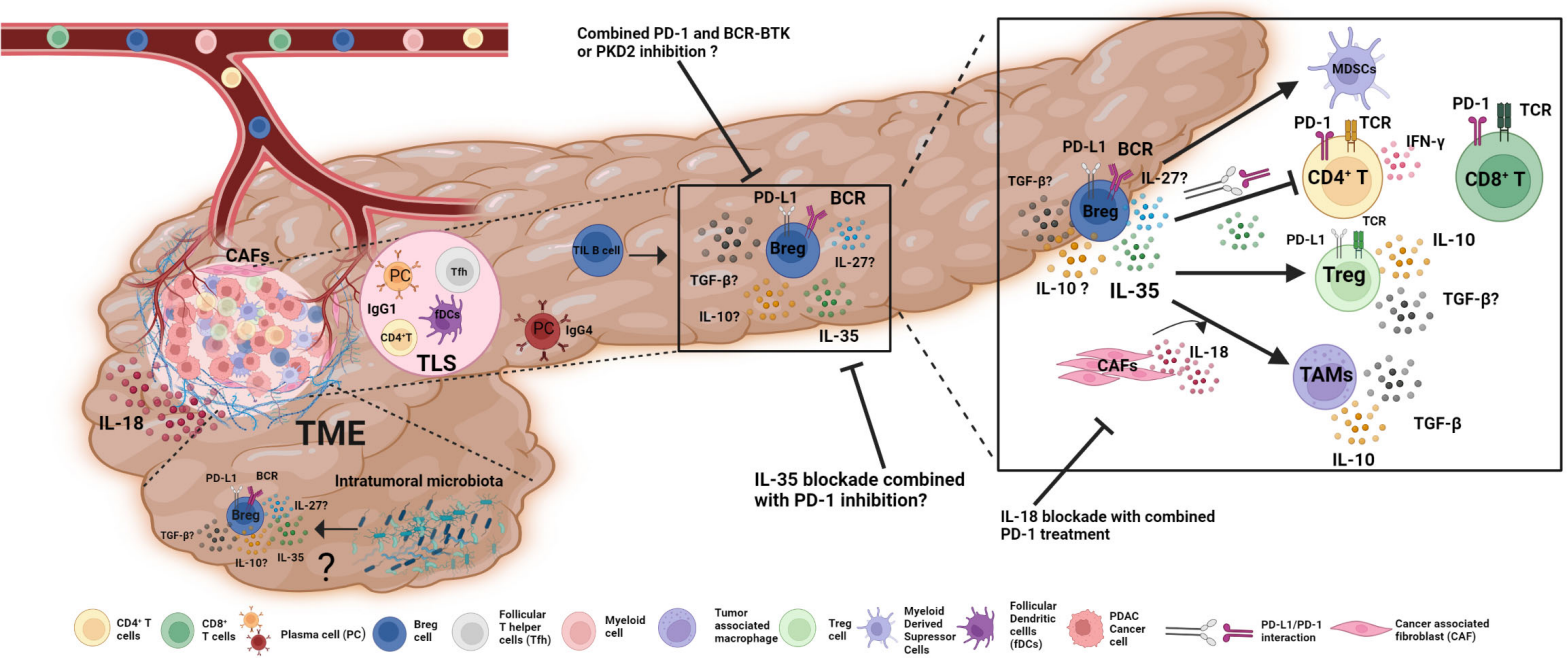
The role of Bregs in PDAC TME and potential approaches for Breg-targeted immunotherapies. Immune cells enter PDAC through the bloodstream and develop anti- or pro-tumorigenic characteristics. B cells are highly infiltrated PDAC-TME and show dual roles. In tertiary lymphoid structures (TLS); naive B cells that detect tumor-associated antigens differentiate into plasma B cells with the aid of Tfh and fDCs and produce IgG1 to exhibit anti-tumoral immune functions. However, plasma cells scattered within the TME produce an anti-inflammatory antibody, IgG4. Bregs are the most prominent B cell subset in PDAC TME, secreting anti-inflammatory cytokines IL-35 and IL-10 that boost Tregs, myeloid-derived suppressor cells (MDSCs), and tumor-associated macrophages (TAMs) while restricting the activity of effector CD4^+^ and CD8^+^ T cells. Bregs, together with their surface-bound PD-L1, interact with PD-1 on the surface of T lymphocytes to decrease their effector activity. IL-18 production from cancer-associated fibroblasts (CAFs) influences Breg development and function in TAMs. The effects of other factors, such as intra-tumoral microbiota, on Breg differentiation and function, remain unclear. In addition to current treatment options; Breg-targeted immunotherapies, such as a combination of BCR-BTK signaling inhibition or IL-18 and IL-35 blockade with immune checkpoint inhibitors, might be promising approaches for PDAC immunotherapy in addition to existing alternatives.

B lymphocytes display dual roles in the tumor microenvironment, which can be either anti-tumorigenic or pro-tumorigenic (21). Tumor-infiltrating B cells maturate and differentiate into IgG1-producing plasma cells and memory B cells in ectopic lymph nodes like tertiary lymphoid structures (TLS) with the help of follicular helper Tfh cells (2, 20, 22) (**Figure 1**). Memory and plasma B cells in TME respond to tumor-associated antigens by producing IgG1 and IgE antibodies that activate the complement system, phagocytosis, or antibody-dependent cytotoxicity of NK cells and macrophages (23–25). Within TLS, B cells can present tumor-associated antigens to T cells (26). Despite this, pro-tumorigenic B cells promote tumor growth and progression by producing circulating immune complexes (CICs), suppressing anti-tumor immune functions, and promoting angiogenesis (2, 27, 28).

Regulatory B cells (Bregs) exhibit anti-inflammatory properties that help to maintain host tolerance. The origin and development of Bregs are still unclear but many Breg subsets were discovered in mice and humans. While in mice mainly CD19^+^CD21^hi^CD23^hi^CD24^hi^ transitional 2 marginal-zone precursor cells (T2-MZP), IL-10 producing CD1d^hi^CD5^+^ B10 cells, CD19^+^CD21^hi^CD23-marginal-zone (MZ) B cells, CD19^+^ Tim-1^+^ B cells, CD19^+^CD5^+^ B1a cells, CD9^+^ B cells, CD138^+^CD44^hi^ plasma B cells, CD44^hi^ B cells, and CD138^+^CD44^hi^ plasmablasts are identified as Breg subsets; in humans, CD19^+^CD24^hi^ CD38^hi^CD1d^hi^ B cells, CD24^hi^CD27^+^ B10 cells, CD19^+^ Tim-1^+^ B cells, CD19^+^CD24^hi^CD27^+^ B10 cells, CD9^+^ B cells, CD19^+^CD38^+^CD1d^+^IgM^+^CD147^+^ Granzyme B^+^ B cells, CD19^+^CD25^hi^CD71^hi^CD73^−^Br1 cells, CD19^+^CD24^hi^CD38^hi^CD1d^hi^ immature B cells, and CD19^+^CD24^hi^CD27^int^ plasmablasts were described as Breg subsets (29–38).

Bregs contribute to the reduction of inflammation in autoimmune and infectious disorders, as well as the attenuation of anti-tumor immune responses in several cancer types that promotes tumor growth and metastasis (39). Breg cells affect immune cells such as T cells and NK cells or cancer-associated fibroblasts (CAFs) by secreting anti-inflammatory IL-10, IL-35, and TGF-β or by expressing cell-membrane bound immune-checkpoint molecules, including PD-1, PD-L1, and granzyme B (GrB) in solid tumors (2, 36). In addition to Breg, PD-L1 expression was upregulated in naive B cells and PCs. The CD19^+^PD-L1^+^ CD138^+^ IgA^+^ IL-10^+^ PC subsets in both human and murine prostate cancer models produce IL-10 and IgA and resist chemotherapy (40). Moreover, CD19^+^PD-L1^+^CD80^+^ CD86^+^ MHC-II^+^CD44^+^ CD69^+^ naive B cells play suppressive roles in Lewis lung cancer by inhibiting Th17 cells *via* the PD-1/PD-L1 pathway (41). Recent research has focused on the role of Bregs in pancreatic cancer, which promotes T- cell exhaustion and activates MDSCs, Tregs, and TAMs in solid tumors (42–45). Furthermore, greater Breg levels in the peripheral blood of PDAC patients correlated with worse overall survival. To investigate the involvement of B cells in PDAC progression, pancreatic cancer cell lines, human peripheral blood & pancreatic tissue samples, xenografts, or genetically-engineered murine models (KPC, KC, and orthotopic) have been utilized (13, 20, 46, 47).

### 2.1 B -lymphocytes in pancreatic cancer murine models

In terms of having a similar development pattern to PDAC and comparable amounts of infiltrating B cells in humans, the KPC mouse (KrasG12D/+; Trp53R172H/+; Pdx-1-Cre) is the gold standard of PDAC preclinical models (13). The majority of infiltrated B cells in KPC tumors are memory B-cells, which are activated and produce a significant amount of immunoglobulins, whereas Bregs and plasma cells are at lesser levels (20, 48).

The KC mouse (KrasG12D/+; Pdx-1- Cre) expresses solely mutant Kras and consequently develops PDAC more slowly (47). Importantly, the proportion of CD19^hi^CD1d^hi^CD5^+^ Breg cells in the KC mouse model was greater than in wild-type mice. In the KC mouse model, where B cells infiltrate less into pancreatic tissue, the frequency of B2 cells decreases while the frequency of B1 cells increases within the tumor, as compared to wild-type mice (48). KC mouse models exhibited lower immunogenicity than KPC mice, suggesting that the KC mouse model is more appropriate to describe B cell involvement in PDAC development rather than PDAC progression (20).

Orthotopic murine models, developed by injecting syngeneic tumor cells from primary pancreatic cancers into wild-type mice, allow researchers to study the role of human B-lymphocytes in tumor development (46).

### 2.2 Bregs in the PDAC microenvironment

In pancreatic ductal adenocarcinoma tissue, B-lymphocytes are located within TLS or scattered in tumor-infiltrating T lymphocytes-enriched (TILs) stroma (49). Tumor-infiltrating B (TIL-B) cells and plasma B cells (PCs), which are scattered within the tumor stromal microenvironment, exert immunosuppressive effects. In PDAC, these PCs produce anti-inflammatory IgG4 and dampen anti-tumoral immune responses (**Figure 1**). Furthermore, tumor-infiltrating B lymphocytes in PDAC can differentiate into IL-10- or IL-35- producing Breg cells with the help of other immune cells such as Tregs and MDSCs, cytokines (IL-18, IL-35), CAFs, tumor-associated antigens, damage-associated molecular patterns (DAMPs), hypoxia, pancreatic microbiota, and metabolites in the tumor microenvironment (7, 21, 36, 50, 51).

A high number of IL-10 and/or IL-35-producing Bregs are observed in the tumor stroma of PDAC murine models (KPC and KC) in addition to PDAC patient samples (**Table 1**). These Bregs are described as IL-10-producing CD1d^hi^CD5^+^ B (B10) or IL-35-producing CD19^+^CD21^hi^CD1d^hi^CD5^+^ B cells in mice and IL-35-producing CD19^+^CD24^hi^CD38^hi^ B cells in humans. As of yet, no other regulatory B cell subsets have been described in PDAC in mice or humans (25, 51–55).

**Table 1:** Bregs in the PDAC.

Mice Model	Human	Key Features	Reference
KC model	PanIN lesions	In mice CD1d^hi^CD5^+^ expressing B cells, produce IL-35; In human PanIN lesions B-cell specific p35 expression	Gupta et al, 2016 (25)
C57BL/6J mice with murine PC cell line Panc02 cells or LTPA cells	Peripheral blood	Both in mice and PDAC patients CD19^+^ IL-10^+^ B cells; increased expression of PD-L1 *via* IL-18/IL18R signaling	Zhao et al., 2018 (51)
KC model	–	CD1d^hi^CD5^+^ expressing B cells, produce IL-10 and IL-35; increased stromal CD8^+^IFNγ^+^ cytotoxic T cells with BTK signaling	Das et al,2019 (52)
KPC and KC model	Intratumoral Breg cells; PDAC patient’s lesions	In mice increased IL-35-producing Breg cells with an elevated level of CD8^+^ T cell infiltration; In humans IL-35 producing CD19^+^CD24^hi^CD38^hi^ Bregs	Mirlekar et al, 2018 (53); Mirlekar et al, 2020 (54)
KPC4662 tumor-bearing mice	–	IL-35 producing CD19^+^CD1d^hi^ CD21^hi^ CD5^+^ Bregs	Michaud et al.,2021 (55)

The secreted protein IL-18 promotes CD19^+^PD-L1^+^IL-10^+^ Breg differentiation and enhances immunological tolerance, which leads to the development and metastasis of PDAC (51). In addition to IL-18, other major secreted molecules include chemokines such as CXCL13 and CCL21, which are responsible for B-cell migration and accumulation within tumors (**Figure 1**). Aside from the well-known involvement of HIF1-α in solid tumor progression in various cancer types, HIF1-α deficiency in human pancreatic tumor samples and KC murine models resulted in increased secretion of CXCL13 and CCL21, which induces accumulation of CD19^+^ B cells in TME and leads to PDAC development (47).

The pancreas was known to be devoid of microorganisms. Several findings, however, confirmed the presence of bacteria, irrespective of the disease status. The role of microbiota and microbial particles within the tumor microenvironment has attracted increasing attention concerning PDAC development (10, 11, 55). In the KPC mouse model, the PDAC microbiome induces immune tolerance in the tumor microenvironment *via* macrophage programming, which is dependent on TLR ligation (10). Also, recently, in the KPC mouse model, LPS (lipopolysaccharide, TLR4 agonist) stimulation leads to an increase in IL-35 expression from CD19^+^CD1d^hi^CD21^hi^CD5^+^ Bregs, which are the main IL-35-producing subset in PDAC (55). This raises the question of whether certain bacteria can contribute to PDAC development.

Bregs inhibit the functions of CD4^+^ effector T and CD8^+^ cytotoxic T cells, macrophages, DCs, and NK cells in solid tumors by secreting IL-10, IL-35, and TGF-β, as well as interacting through their cell membrane-bound molecules such as PD-1 and PD-L1 (2). In PDAC, the production of B cell-mediated IL-35 promotes tumorigenesis by inhibiting the CD8^+^ T cell infiltration *via* downregulation of the chemotactic receptors CXCR3 and CCR5, as well as the secretion of the effector cytokine IFN-γ. Interestingly, in PDAC patients, the CD24^+^CD38^+^ B cell subset that secreted IL-35 was more frequent, indicating disease-induced proliferation of this cell subtype (54). In addition to secreting IL-35, regulatory B cells can decrease CD8^+^ T cell response *via* PD-L1-PD-1 interaction. TIL B cells had significantly higher PD-L1 expression levels than peripheral blood (PB) B cells from the same patients. Moreover, PD-L1 levels were considerably greater in PB B cells from stage III and stage IV PDAC patients than in PB B cells from healthy controls. This points to a novel immunoregulatory mechanism in pancreatic cancer. Nevertheless, the localization of these PD-L1^+^ B cells in the TME, as well as their impact on other types of effector cells (such as CD4^+^ T cells and NK cells), has to be investigated further (12). Additionally, the underlying reasons for increased PD-L1^+^B cell frequency in pancreatic cancers are unknown. Yet, it is suggested that the higher PD-L1 expression level in PDAC patients is due to an increase in B cell activation induced by both direct translocations of microbial materials and indirect microbiome-mediated activation of T cells. Investigating the association between the presence of bacteria in PDAC and B cell-mediated PD-L1 expression will enable us to determine whether intratumoral bacteria can impact anti-tumor treatment by boosting B cell-mediated immune suppression (12).

Given the pro-tumorigenic activities of B cells in PDAC, B-cell targeted immunotherapy has emerged as a potential target possibility. The following section will cover current B-cell targeted therapies focusing on Breg cells.

## 3 Breg cell therapies for PDAC

During the last decade of study, B lymphocytes have acquired importance in tumor development and progression. B cell-targeted immunotherapeutic approaches consist of monoclonal antibodies, inhibiting or depleting B cells, activated anti-tumoral B cells, vaccines, and targeting tumor-associated auto-antibodies in esophagus cancer, melanoma, colorectal cancer, breast cancer, and ovarian cancer (2, 56, 57). However, little is known regarding Breg cell therapy for the treatment of pancreatic cancer. According to recent research, the IL-35-producing Bregs are the main players in PDAC progression. We focused on IL-35-producing Bregs and their pro-tumorigenic pathways in this mini-review, which may provide novel approaches for Breg cell therapies against PDAC.

Through BCR signaling, regulatory B cells produce IL-35, which suppresses CD4^+^ T cell responses *in vivo*, promoting pancreatic cancer progression (25, 53, 55). Therefore, B-cell therapeutic approaches may benefit from focusing on downstream components of BCR signaling. CRT0066101, a PKD1/2 (Protein Kinase D1/2) inhibitor, is now being utilized to treat colon and pancreatic cancer. In the mouse PDAC microenvironment, PKD2 is a critical regulator of IL-35 expression in CD19^+^CD1d^hi^CD21^hi^CD5^+^ Bregs. Treatment of the KPC4662-cell orthotopic PDAC mouse model with PKD1/2 inhibitor (CRT0066101) leads to a reduction in PDAC tumor volume (55).

Bruton’s tyrosine kinase (BTK) signaling is critical for B cell development and function since it induces tumorigenic IL-35 secreting CD1d^hi^CD5^+^ Bregs in the KC-PDAC model. Tirabrutinib, which is a BTK inhibitor, specifically decreases percentages of Bregs in PDAC-TME while not affecting stromal CD19^+^ B cells (52). Even though, clinical trials (NCT02362048) for the BTK inhibitor acalabrutinib monotherapy or the combination of acalabrutinib and anti-PD-1 blockade pembrolizumab reduced MDSCs; no significant clinical activity was demonstrated in patients with advanced pancreatic ductal adenocarcinoma (https://beta.clinicaltrials.gov/). Combining BTK inhibitors with IL-35 inhibition can improve anti-tumoral immune responses for PDAC immunotherapies (**Figure 1**).

IL-18 is expressed in epithelial and myeloid cells with a dual role in tumor progression and suppression. IL-18 has been detected in TME of patients with esophageal squamous cell carcinoma, pancreatic cancer, breast cancer, lung cancer, renal cell carcinoma, multiple myeloma, hepatocellular carcinoma, and oral cavity cancer (58–60). Patients with PDAC and murine pancreatic models revealed that CD19^+^PD-L1^+^IL10^+^IL18R^+^ Breg subsets produce IL-10 and inhibit antibody-dependent cytotoxicity of T- and NK cells by pancreatic cancer-derived IL-18 (51). Tumor development and metastasis were suppressed in mouse models when both IL-18 inhibitor and PD-1/PD-L1 inhibitor were coupled. However, no clinical trials for this have been conducted thus far (51). Suppression of IL-18 and PD-1/PD-L1 in PDAC patients may pave the way for future PDAC immunotherapies (61, 62).

PDAC TME-resident stroma cells, produce CXCL13 to recruit the murine CD1d^hi^CD5^+^ Breg subset, which promotes tumor growth by producing IL-35, in the tissue. B-cell-specific IL-35 expression was detected in murine samples as well as human PanIN lesions (25). In comparison, the combination of anti-PD-1 and B cell-specific loss of IL-35 resulted in an additional reduction in tumor growth in the KPC mouse model by augmenting the CD4^+^ effector T cell response, decreasing intratumoral Treg frequency, and increasing effector CD8^+^ T cell infiltration and IFN-γ expression (54). However, no clinical trials with patients have been done so far.

The CD40-CD40L signaling pathway promotes antigen presentation by B cells. CD40 B cell vaccination is utilized in patients with renal cell carcinoma and metastatic melanoma by fusing tumor cells with allogeneic B cells from healthy donors’ PBMCs (63). Despite this, no research on B-cells in pancreatic cancer vaccination has been reported.

The role of gut bacteria in pancreatic cancer etiology is becoming more widely acknowledged. The PDAC tumor microbiome’s variety and composition may influence immune infiltration, which in turn influences PDAC survival. Three tumor bacterial taxa, *Sachharopolyspora*, *Pseudoxanthomonas*, and *Streptomyces*, were shown to be considerably enriched in PDAC patients with long-term survival. Saccharopolyspora spp. has been linked to developing a pro-inflammatory milieu that attracts pro-inflammatory cells and increases IFN-γ secretion in inflammatory lung disease. Its involvement in PDAC, however, has yet to be studied. Tumor microbiome sequencing might be relevant in the future for adjuvant research including microbiome therapies (11, 64).

## 4 Discussion

Effective immunotherapeutic responses have been demonstrated to correlate with pre-existing intratumoral effector T-cell infiltration, which is lacking in the vast majority of PDAC patients (65). T-cell immunotherapy is at the forefront of therapeutic approaches for a wide variety of cancers. Although B lymphocytes are highly infiltrated in pancreatic adenocarcinoma, their significance in tumor formation has been underappreciated. B lymphocytes have two distinct roles in the PDAC tumor microenvironment, acting either as anti- or pro-tumorigenic (21). Regulatory B cells are known for modulating immune responses during inflammation and autoimmunity by secreting anti-inflammatory cytokines and interacting with other immune cells *via* cell membrane-bound proteins. Their critical function in tumor development, however, has only lately been discovered. Therefore, determining the involvement of Bregs in PDAC is becoming increasingly appealing for developing innovative immunotherapeutic methods.

Currently, downstream BCR signaling molecules (PKD2 or BTK), tumor cell-derived IL-18 cytokine, and Breg-cell-mediated IL-35 are utilized to target pro-tumorigenic suppressor B cells in PDAC. Combined anti-PD-1 therapies with BTK inhibitor acalabrutinib have been used in clinical trials of PDAC patients and anti-PD-1 therapy combined with B cell-specific loss of IL-35 has been tested on mouse models. In the KPC mouse model, a combination of anti-PD-1 and B cell-specific lack of IL-35 reduced tumor development. In the Panc02-lucifer-cell- orthotopic PDAC mouse model, the pairing of IL-18 inhibitor with PD-1/PD-L1 blockade inhibited tumor development and metastasis. However, acalabrutinib in combination with anti-PD-1 therapy did not demonstrate significant clinical effects in PDAC patients. Overall, these findings indicate that B cell-based immunotherapy, like combining immune checkpoint blockades with inhibition of BTK, IL-18, or IL-35, offers a promising alternative strategy to target PDAC development. Furthermore, given the recent discovery of intratumoral microbiota in PDAC, tumor microbiome sequencing might be beneficial for cancer prognosis, and fecal microbiota transplantation is emerging as a potential therapeutic approach to increase the lifespan of PDAC patients. Nevertheless, further research is required to fully understand the role of regulatory B cells in this lethal cancer to target them in immunotherapeutic approaches.

## Author contributions

AS-Y took the lead in design and conception of the article and in writing the manuscript, wrote the manuscript with input from all authors. ZS, IA, and BD contributed to conception and design, wrote sections of the manuscript. ZS prepared the figure and the table. All authors contributed to the article and approved the submitted version.
